# Equity assessment of the distribution of CT and MRI scanners in China: a panel data analysis

**DOI:** 10.1186/s12939-018-0869-y

**Published:** 2018-10-05

**Authors:** Luyang He, Hao Yu, Lizheng Shi, Yao He, Jingsong Geng, Yan Wei, Hui Sun, Yingyao Chen

**Affiliations:** 1 Department of Hospital Management, School of Public Health, Key Lab of Health Technology Assessment, National Health Commission, Fudan University, Shanghai, 200032 People’s Republic of China; 2 RAND Corporation, Pittsburgh, USA; 3 School of Public Health, Tulane University, New Orleans, USA

**Keywords:** High technology medical equipment, Equity, Gini coefficient, Concentration index

## Abstract

**Background:**

Distribution equity assessment of computed tomography (CT) and magnetic resonance imaging (MRI) scanners is an important dimension of access to health technology. However, limited studies on the subject have been done in China. This study aims to reveal the distribution status of CT and MRI scanners and assess their distribution equity of them in China.

**Methods:**

Five provinces with 66 cities from China were selected as the study sites. Descriptive analysis was used for the absolute number and number per million population of CT and MRI scanners in the study sites. Fixed effect model was used to examine the health service factors that were associated with the allocation of CT and MRI scanners. The Gini coefficient and concentration index was used to evaluate the distribution equity of CT and MRI scanners.

**Results:**

The absolute number and number per million population of CT and MRI scanners in five provinces were lower than those of Organization for Economic Co-operation and Development (OECD) countries, but annual growth rates were relatively higher from 2005 or 2006 to 2013. Population, GDP, number of hospitals, number of health professionals, number of hospital beds, number of outpatient visits, and number of inpatient visits all had a positive correlation with the allocation number of CT and MRI scanners. Moreover, the number of health professionals and the number of beds had a much closer correlation than other variables. All the Gini coefficients of CT and MRI had decreased overall. The concentration indices of CT and MRI were all positive and no more than 0.30.

**Conclusions:**

Large gaps in the number of CT and MRI scanners per million population between China and OECD countries emerge, although the growth rate is higher in China. The distribution equity of CT and MRI scanners in China was relatively good from 2005 or 2006 to 2013. The overall distribution equity of CT and MRI scanners also improved during the period. However, consideration attention should be given to the area with large economic disparities.

## Background

Computed tomography (CT) and magnetic resonance imaging (MRI) scanners are now common medical equipment in China and play a highly important role in the diagnosis of diseases. CT and MRI scanners belong to the category of Type-IIHigh Technology Medical Equipment (HTME), and are managed by the national and provincial health departments according to the *Management Methods on Allocation and Utilization of High Technology Medical Equipment* issued in 2005 by the National Health and Family Planning Commission (NHFPC; formerly the Ministry of Health) [1]. HTME refers to either medical equipment included in the management list of the Health Department under the State Council or equipment not included in the list but priced above 5 million Chinese yuan (CNY) and are allocated at provincial level hospitals for the first time. The Certificate of Need (CON) policy, established as the list went into effect, aims to improve “appropriate allocation and the efficient use of medical equipment” through regional health planning and quota control. The NHFPC controls the total amount of HTME in China, and provincial health departments formulate allocation plans under the quota set by the NHFPC [2]. The NHFPC is responsible for the CON licensure of Type-IHTME, whereas provincial health departments are responsible for that of Type-IIHTME. Hospitals must apply for CON before purchasing HTME. The purchase of HTME can be classified into several categories, including hospital-paid, government reimbursement, government-paid and donation, among which hospital-paid is the main mode.

The quantity of CT and MRI scanners increased rapidly in the late twentieth century. Their allocation have become reasonable after the implementation of the CON policy while problems still exist in the utilization of CT and MRI scanners according to limited research. Yan Wei’s study corroborated that the use quantity and hospitalization utilization rate of CT and MRI scanners has been increasing, but the outpatient utilization rate was relatively stable based on an investigation on 131 hospitals from 2009 to 2013. The hospitalization rate of CT and MRI scanners in secondary hospitals has reached 50.44% and 19.49% which were well above the international level [3]. Research conducted by Shengnan Duan affirmed that the overutilization and underutilization of CT and MRI scanners coexisted in China based on 1573 medical records from 16 hospitals in 2012. The unreasonable layout of equipment was one of the main reasons for underutilization [4]. A similar conclusion was found in the research directed by Jiaqi Liu based on 801 medical records from 8 hospitals in 2013 [5]. The allocation of HTME has a great impact on utilization. Exploring and improving the distribution of CT and MRI scanners to promote their scientific utilization is necessary.

Some studies contended that although the number of CT and MRI scanners per million population in China was lower than medians in OECD countries, the growth rate was higher in China than most OECD countries [2, 6]. The numbers of CT and MRI scanners in secondary and tertiary hospitals in China were 12,888 and 6762 respectively in 2015 according to statistics from the Chinese Medical Doctor Association (CMDA). With the widespread use and increasing number of CT and MRI scanners in China, macro research has become more important to provide evidence for the improvement of the management policy. However, few studies could provide an overview of CT and MRI scanners in China. Three gaps are identified in the macro research on CT and MRI scanners in China.

First, inconsiderable research evidence validates the distribution trend of CT and MRI scanners in China. Some studies have tried to affirm the distribution of CT and MRI scanners in China to some extent [7–11]. Owing to limited provinces or short period, we cannot obtain a clear overview.

Second, only a few studies explore the correlation between health service indicators and the distribution of CT and MRI scanners in China. Studies on the determinants of the distribution of HTME have been conducted by researchers from other countries, but few have been done in China. While some studies have analyzed the influence of population and GDP on the distribution of CT and MRI scanners, few studies have analyzed the influence of health service indicators [2, 12].

Third, inconsiderable research reveals the changing trend of the distribution equity of CT and MRI scanners in China. Previous studies focus on their equity in a certain region or a short period. The improvement or setback of distribution equity cannot be recognized clearly in China, especially after the implementation of the CON policy [13–18].

This study tries to describe the allocation of CT and MRI scanners in China after the implementation of the CON policy. It also aims to conduct preliminary investigation on the influence of health service indicators on the distribution of CT and MRI scanners in China. Given that OECD consists of developing or developed countries and may represent the global average allocation number of CT and MRI scanners, the comparison of the numbers of CT and MRI per million population is made between China and the OECD. We will also evaluate equity as an important aspect of the appropriateness of CT and MRI scanner distribution as well as the changing trend of distribution equity after the implementation of the CON policy. We hope that this study will fill the gaps in the current evidence base to some extent.

## Methods

### Sample and data collection

Sixty-six cities in five provinces in China were selected as the study sites in our study, including Zhejiang, Guangdong, Hunan, Shanxi, and Shaanxi Provinces. Zhejiang and Guangdong are in East China and represent provinces with advanced socioeconomic development in China. Hunan and Shanxi are in the central region of China and represent medium socioeconomic development in China. Shaanxi is in West China and represent low socioeconomic development in China.

Data were collected from the provincial health department in each of the five provinces. The demographic and socioeconomic data of sixty-six cities within the five provinces were obtained from the Statistical Yearbook of each province from 2005 to 2013. The numbers of CT and MRI scanners from 2005 to 2013 and other health system data including the number of hospitals, number of beds, number of health professionals, number of inpatient visits, number of outpatient visits from 2009 to 2013 were gathered through a questionnaire addressed to the provincial health department. Five questionnaires filled out by the five provincial health departments were collected via mail. Owing to data loss, the numbers of CT and MRI scanners of Shanxi in 2005 and Shaanxi in 2005, 2007, and 2008 were not included in the analysis.

### Statistical analysis

#### Numbers and growth rates of CT and MRI scanners

Descriptive analysis was used for the absolute numbers and numbers per million population of CT and MRI scanners in the study sites and OECD countries. The annual growth rates (AGRs) of the two types of medical equipment were also calculated from 2005 or 2006 to 2013. The formula of AGR is as follows:

$$ \mathrm{AGR}=\sqrt[n]{\frac{B}{A}}-1, $$

where *B* is the quantity of CT or MRI scanners in 2013, *A* is the quantity of CT or MRI scanners in 2005 or 2006, and *n* represents the number of years.

### Influencing effect of health service indicators on the distribution of CT and MRI scanners

Fixed effect model (FE model) was conducted to deepen our analysis based on the Hausman test and F test [3]. The dependent variable is the absolute numbers of CT and MRI scanners, and the independent variables consist of the population, GDP, number of hospitals, number of health professionals, number of hospital beds, number of outpatient visits, and number of inpatient visits of a given city [12, 19–21]. Given the strong self-correlation among all health service indicators, they were included in the model separately with socioeconomic variables. The Fixed effect model in this study could be expressed in the following equation where *Y*_*it*_ referred to the number of CT or MRI scanners, *X*_*k,it*_ to other covariates in the model, *a*_*i*_ to the intercept and μ_it_ to error term. Log transformation was used to cope with the abnormal distribution of the number of CT or MRI scanners. *Y*_*it*_ *+ 1* was transformed for the number of CT or MRI scanners since the min value of that is 0. STATA 13.0 was used to conduct the analysis.

$$ \ln {Y}_{it}=\ln {\beta}_1{X}_{1,\mathrm{it}}+\ln {\beta}_2{X}_{2,\mathrm{it}}+\dots +\ln {\beta}_k{X}_{k,\mathrm{it}}+{a}_i+{\mu}_{it} $$

$$ \ln \left({Y}_{it}+1\right)=\ln {\beta}_1{X}_{1,\mathrm{it}}+\ln {\beta}_2{X}_{2,\mathrm{it}}+\dots +\ln {\beta}_k{X}_{k,\mathrm{it}}+{a}_i+{\mu}_{it} $$

### Equity assessment on the distribution of CT and MRI scanners

The Gini coefficient and concentration index were used to evaluate the equity of the distribution of CT and MRI scanners. The Gini coefficient was primarily developed and used in economics, but now it has also been widely recognized in public health and epidemiology. The Gini coefficient takes on the value from 0 to 1. According to the conception of the Gini coefficient, the bigger the value, the more inequitable the distribution. A Gini coefficient that is smaller than 0.2 indicates a very low inequity level, and one that is bigger than 0.4 means a high inequity level [22, 23]. The following formula was employed to calculate the Gini coefficient:

$$ \mathrm{G}=\sum \limits_{i=1}^n{P}_i{Y}_i+2\sum \limits_{i=1}^{n-1}{P}_i\left(1-{V}_i\right)-1, $$

where *P*_*i*_ is the cumulative proportion of the population in each group; Y_i_ is the cumulative proportion of the health resources in each group; and *V*_*i*_ *= Y*_*1*_ *+ Y*_*2*_ *+ ……Y*_*i*_; *i* is the fractional rank in terms of the number of CT and MRI scanners from the lowest to the highest number.

The concentration index was introduced to measure the inequity of health and medical services in different socioeconomic conditions by the World Bank. It could quantify the level of inequity of the distribution of health resources relevant to the economy. Its value ranges from − 1 to 1. The bigger the absolute value, the more inequitable the distribution. 0 means absolute equity. The concentration index could also reflect the direction of the distribution of the resource. A positive value indicates that resources gather in the richer area, while a negative value means that resources gather in the poorer area [24–26]. The following formula.

was employed to calculate the concentration index:

$$ \mathrm{S}=\frac{1}{2}\sum \limits_{i=0}^{n=1}\left({Y}_i+{Y}_{i+1}\right)\left({X}_{i+1}-{X}_i\right), $$

where *Y*_*i*_ is the cumulative proportion of CT and MRI scanners, *X*_*i*_ is the cumulative proportion of population, and *i* is the fractional rank according to per capita GDP beginning with the lowest; and CI represents the concentration index.

STATA13.0 with the Distributive Analysis Stata Package (DASP) was used to calculate the Gini coefficient and concentration index. DASP is mainly designed to assist researchers and policy analysts who are interested in conducting distributive analysis with Stata [27].

## Results

### Numbers of CT and MRI scanners in the study sites

Table 1 shows the distribution and average AGR of CT and MRI scanners in the five provinces from 2005 or 2006 to 2013. The numbers of CT and MRI scanners had both been increasing during the period, while the number of MRI scanners grew faster than that of CT scanners. Overall, the numbers of CT and MRI scanners in Zhejiang, Guangdong, and Shaanxi were larger than those in Hunan and Shanxi, and so were the average AGRs that were 12.01%, 11.69%, and 11.78%, respectively, versus 3.11% and 6.96%,respectively, for CT scanners, and 15.40%, 16.03%, 22.94%, respectively, versus 11.61% and 11.50%, respectively, for MRI scanners.

**Table 1:** Numbers of CT and MRI scanners in 5 provinces

	Province	2005	2006	2007	2008	2009	2010	2011	2012	2013	AGR
CT	Zhejiang	138	178	207	230	266	302	343	378	429	12.01%
	Guangdong	239	265	287	384	445	553	615	675	722	11.69%
	Hunan	299	334	342	344	352	359	369	386	406	3.11%
	Shanxi		163	168	172	176	185	230	240	261	6.96%
	Shaanxi		194			321	350	376	398	423	11.78%
MRI	Zhejiang	37	47	60	71	74	86	106	125	155	15.40%
	Guangdong	66	71	83	101	106	153	199	249	292	16.03%
	Hunan	48	58	66	70	78	87	98	111	144	11.61%
	Shanxi		35	39	39	42	46	61	70	75	11.50%
	Shaanxi		49			81	107	135	167	208	22.94%

Table 2 shows the changing trend of the numbers of CT and MRI scanners per million population in the five provinces from 2005 or 2006 to 2013. While the numbers had been increasing across all five provinces, the numbers in Zhejiang, Guangzhou, and Shaanxi were higher than those in Hunan and Shanxi during the period. The annual growth rates were 10.93%, 10.07%, and 11.50% versus 2.53% and 5.85% for CT scanners, and 14.29%, 14.35%, and 22.64% versus 10.99% and 10.35% for MRI scanners. The numbers of CT and MRI scanners per million population in Shaanxi grew the fastest.

**Table 2:** Numbers of CT and MRI scanner per million population in 5 provinces and OECD countries

	Province	2005	2006	2007	2008	2009	2010	2011	2012	2013	AGR
CT	Zhejiang	2.76	3.51	4.02	4.41	5.04	5.54	6.28	6.90	7.80	10.93%
	Guangdong	2.60	2.81	2.97	3.88	4.39	5.30	5.85	6.37	6.78	10.07%
	Hunan	4.73	5.27	5.38	5.39	5.49	5.46	5.59	5.81	6.07	2.53%
	Shanxi		4.83	4.95	5.04	5.14	5.18	6.40	6.65	7.19	5.85%
	Shaanxi		5.24			8.61	9.37	10.05	10.60	11.24	11.50%
MRI	Zhejiang	0.74	0.93	1.16	1.36	1.40	1.58	1.94	2.28	2.82	14.29%
	Guangdong	0.72	0.75	0.86	1.02	1.05	1.47	1.89	2.35	2.74	14.35%
	Hunan	0.76	0.91	1.04	1.10	1.22	1.32	1.49	1.67	2.15	10.99%
	Shanxi		1.04	1.15	1.14	1.23	1.29	1.70	1.94	2.07	10.35%
	Shaanxi		1.32			2.17	2.86	3.61	4.45	5.53	22.64%
Descriptive statistics of OECD Countries											
CT	Mean	17.02	18.42	18.85	21.92	20.39	20.68	23.72	21.91	22.94	3.03%
	Median	11.96	13.09	14.09	14.61	14.99	15.70	16.15	16.79	17.71	4.00%
	Min	3.36	3.40	3.84	4.02	4.14	4.69	4.52	5.10	5.32	4.70%
	Max	51.54	56.72	36.95	96.97	39.14	43.07	101.25	50.51	53.68	0.41%
MRI	Mean	9.22	8.97	9.87	11.34	11.14	12.20	12.68	12.46	13.61	3.97%
	Median	5.40	5.71	7.19	8.57	8.68	9.27	9.55	9.83	11.32	7.68%
	Min	1.33	1.38	1.67	1.62	1.85	1.90	1.95	2.17	2.07	4.52%
	Max	40.14	26.58	25.93	42.96	25.15	31.52	46.86	34.44	35.48	−1.23%

Table 2 presents the descriptive statistics for the distribution of CT and MRI scanners in OECD countries. Up to 2013, the mean and median numbers of CT scanners per million population in OECD countries were 22.94 and 17.71 respectively, larger than those of the five provinces in China. The values were same for MRI scanners, which were 13.61 and 11.32 in OECD countries.

### Influencing effect of health service indicators on the distribution of CT and MRI scanners

Table 3 shows the description statistics of the main variables of sixty-six cities in the five provinces from 2009 to 2013. Owing to the different levels of socioeconomic development in the eastern, central, and western regions, the values of the variables varied greatly.

**Table 3:** Description statistics of main variables

Variable	Median	Mean	SD	Min	Max
N of CT	23.00	28.54	22.09	6.00	167.00
N of MRI	6.00	9.12	9.46	0.00	84.00
Population (10,000)	385.71	434.36	220.57	83.31	1292.68
GDP (billion CNY)	111.82	190.35	230.31	15.44	1542.01
N of hospitals (100)	57.00	71.04	52.94	12.00	281.00
N of Health professionals (100)	124.53	164.79	142.14	21.04	819.22
N of Beds (100)	108.45	138.29	113.09	19.76	648.64
N of outpatient visits (10,000)	371.87	837.89	1435.81	4.41	8735.53
N of inpatient visits (10,000)	28.24	37.13	31.88	4.41	205.42

*SD* standard deviation, *N* number, *Min* minimum, *GDP* gross domestic product

Tables 4 and 5 exhibit the results of the panel regression analysis. Five different health service indicators are included in five regression models respectively combined with population and GDP for CT and MRI scanners. We corroborated that adding the number of health professionals to the model had the largest power to predict the distribution of CT and MRI scanners according to the value of R^2^, and then the number of beds, number of hospitals, number of inpatient visits and number of outpatient visits.

**Table 4:** Results of panel regression analysis for CT scanners distribution

Variables	Model 1	Model 2	Model 3	Model 4	Model 5
Population	0.95^*^	0.87 ^*^	0.88 ^*^	0.90 ^*^	0.86 ^*^
GDP	0.49^**^	0.32 ^**^	0.45 ^**^	0.41 ^**^	0.41 ^**^
Hospitals	0.11				
Health professionals		0.43^**^			
Beds			0.13		
Outpatient visits				0.17^**^	
Inpatient visits					0.14 ^*^
Constant	−6.47^**^	−8.34 ^**^	−6.49^**^	−6.10^**^	−5.40 ^*^
Model	FE Model	FE Model	FE Model	FE Model	FE Model
F test	*p* < 0.01	*p* < 0.01	*p* < 0.01	*p* < 0.01	*p* < 0.01
Hausman test	*p* < 0.01	*p* < 0.01	*p* < 0.01	*p* < 0.01	*p* < 0.01
R^2^	0.37	0.43	0.38	0.29	0.38

^*^p < 0.05; ^**^p < 0.01

**Table 5:** Results of panel regression analysis for MRI scanners distribution

Variables	Model 1	Model 2	Model 3	Model 4	Model 5
Population	−0.30	−0.57	−0.67	−0.51	−0.91
GDP	1.01 ^**^	0.70 ^**^	0.75^**^	0.81 ^**^	1.04 ^**^
Hospitals	0.55 ^**^				
Professionals		0.97^**^			
Beds			0.72 ^**^		
Outpatient visits				0.54^**^	
Inpatient visits					0.005^**^
Constant	−5.62	−8.75 ^*^	−5.95	−3.67	−0.13
Model	FE Model	FE Model	FE Model	FE Model	FE Model
F test	*p* < 0.01	*p* < 0.01	*p* < 0.01	*p* < 0.01	*p* < 0.01
Hausman test	*p* < 0.01	*p* < 0.01	*p* < 0.01	*p* < 0.01	*p* < 0.01
R^2^	0.33	0.43	0.37	0.12	0.22

^*^p < 0.05; ^**^p < 0.01

### Equity assessment across cities within each of the study sites

#### Gini coefficient

Table 6 shows the Gini coefficients of CT and MRI scanners in the five provinces from 2005 or 2006 to 2013. The Gini coefficients of CT scanners decreased from 0.225 to 0.153 in Zhejiang (2005–2013), from 0.336 to 0.226 in Guangdong (2005–2013), from 0.143 to 0.119 in Hunan (2005–2013), from 0.181 to 0.176 in Shanx (2006–2013), from 0.131 to 0.090 in Shaanxi (2006–2013), and from 0.238 to 0.220 across all five provinces (2006–2013). Despite fluctuation, the overall Gini coefficient decreased.

**Table 6:** Gini coefficients of CT and MRI scanners in 5 provinces

	Province	2005	2006	2007	2008	2009	2010	2011	2012	2013
CT	Zhejiang	0.225	0.156	0.125	0.136	0.146	0.124	0.144	0.148	0.153
	Guangdong	0.336	0.318	0.317	0.254	0.217	0.223	0.222	0.233	0.226
	Hunan	0.143	0.117	0.115	0.117	0.111	0.106	0.105	0.106	0.119
	Shanxi		0.181	0.172	0.183	0.175	0.170	0.150	0.156	0.176
	Shaanxi		0.131			0.094	0.089	0.092	0.090	0.090
	Total		0.238			0.221	0.208	0.208	0.213	0.220
MRI	Zhejiang	0.336	0.304	0.284	0.197	0.173	0.202	0.170	0.162	0.155
	Guangdong	0.402	0.375	0.353	0.332	0.309	0.307	0.279	0.317	0.313
	Hunan	0.276	0.292	0.305	0.313	0.282	0.245	0.231	0.227	0.175
	Shanxi		0.298	0.278	0.278	0.319	0.251	0.220	0.267	0.262
	Shaanxi		0.287			0.202	0.128	0.096	0.106	0.091
	Total		0.341			0.311	0.293	0.282	0.306	0.305

**Fig. 1:**
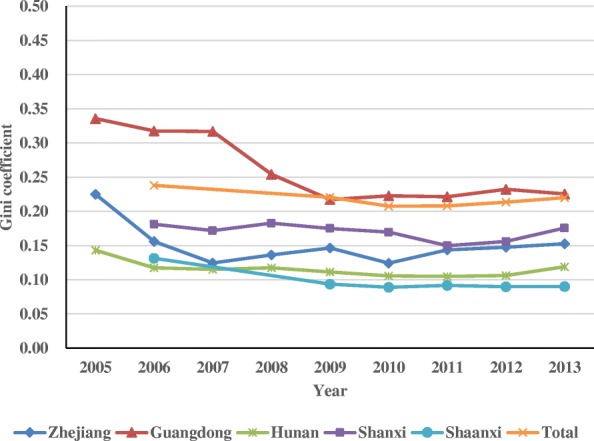
Gini coefficients of CT scanner in the five provinces

**Fig. 2:**
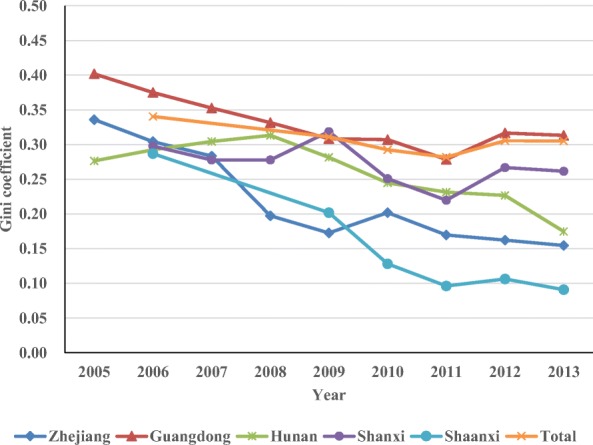
Gini coefficients of MRI scanners in the five provinces

The Gini coefficients of MRI scanners were larger than those of CT scanners in the same year. The Gini coefficients of MRI scanners in Zhejiang decreased from 0.336 to 0.155 (2005–2013), from 0.402 to 0.313 in Guangdong (2005–2013), from 0.276 to 0.175 in Hunan (2005–2013), from 0.298 to 0.262 in Shanxi (2006–2013), from 0.287 to 0.091 in Shaanxi (2006–2013), and from 0.341 to 0.305 across all five provinces (2006–2013). All Gini coefficients affirmed a downward trend overall. Figures 1 and 2 clearly show the changing trend of the Gini coefficients of CT and MRI scanners.

#### Concentration index

**Fig. 3:**
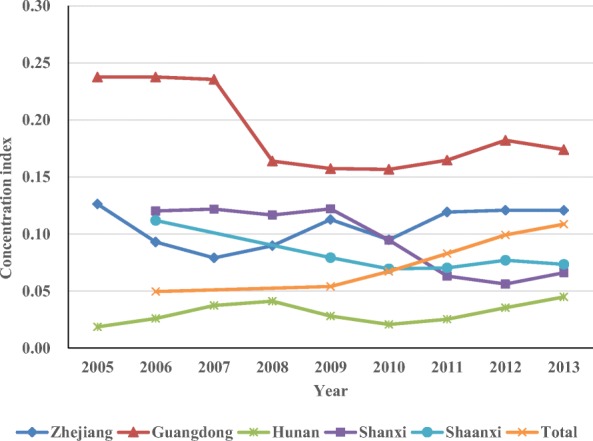
Concentration indices of CT scanners in the five provinces

**Fig. 4:**
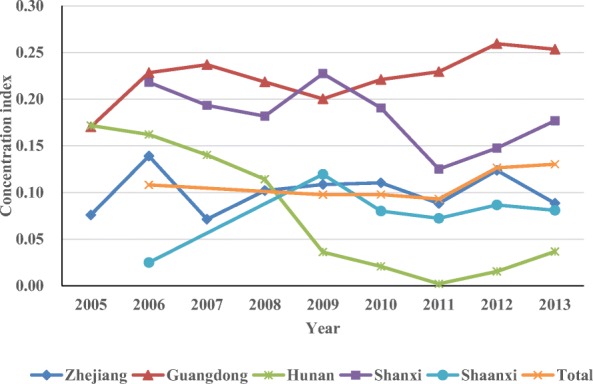
Concentration indices of MRI scanners in the five provinces

Table 7 shows the concentration indices of CT and MRI scanners in the five provinces from 2005 or 2006 to 2013. All concentration indices were positive. The concentration indices of CT scanners in Zhejiang, Guangdong, Shanxi, and Shaanxi confirmed a downward trend, changing from 0.126 to 0.121 (2005–2013), from 0.238 to 0.174 (2005–2013), from 0.120 to 0.066 (2006–2013), and from 0.112 to 0.073 (2006–2013) respectively. However, the concentration index of CT scanners in Hunan and the overall index of the five provinces increased from 0.019 to 0.045(2005–2013) and from 0.050 to 0.109 (2006–2013), respectively. The concentration indices of MRI scanners in Hunan and Shanxi affirmed a downward trend, changing from 0.172 to 0.037 (2005–2013) and from 0.218 to 0.177 (2006–2013) respectively. However, the indices of Zhejiang, Guangdong, Shaanxi and the overall index of the five provinces increased from 0.076 to 0.088 (2005–2013), from 0.170 to 0.109 (2005–2013), from 0.025 to 0.081(2006–2013), and from 0.108 to 0.130 (2006–2013), respectively. Figures 3 and 4 visualize the aforementioned results.

**Table 7:** Concentration index of CT and MRI scanners in 5 provinces

	Province	2005	2006	2007	2008	2009	2010	2011	2012	2013
CT	Zhejiang	0.126	0.093	0.079	0.090	0.113	0.095	0.119	0.121	0.121
	Guangdong	0.238	0.238	0.236	0.164	0.157	0.157	0.165	0.182	0.174
	Hunan	0.019	0.026	0.037	0.041	0.028	0.021	0.025	0.035	0.045
	Shanxi		0.120	0.122	0.117	0.122	0.095	0.063	0.056	0.066
	Shaanxi		0.112			0.079	0.070	0.070	0.077	0.073
	Total		0.050			0.054	0.067	0.083	0.099	0.109
MRI	Zhejiang	0.076	0.139	0.071	0.102	0.108	0.110	0.088	0.124	0.088
	Guangdong	0.170	0.228	0.237	0.218	0.200	0.221	0.229	0.259	0.254
	Hunan	0.172	0.162	0.140	0.114	0.036	0.021	0.002	0.015	0.037
	Shanxi		0.218	0.193	0.182	0.227	0.191	0.125	0.148	0.177
	Shaanxi		0.025			0.119	0.080	0.072	0.087	0.081
	Total		0.108			0.098	0.098	0.093	0.126	0.130

## Discussion

The lack of scientific management and allocation plan, the allocation of HTME lost control, and an arm-race of HTME were set off among hospitals in the late twentieth century. CT and MRI scanners were first introduced in China in 1978 and 1985, respectively. The numbers of CT and MRI scanners have reached 2549 and 356 in 1996 with a high growth rate [28]. The situation has been improved after the CON policy was implemented in 2005. Increase in quantity has become more reasonable according to data analysis, especially in the central regions with a relatively low AGR from 2005 or 2006 to 2013. However, the proportion of the income from the examination and treatment of HTME in public hospitals has been increasing. The unreasonable allocation and utilization are the major reasons for the problem. *Several Suggestions on Controlling the Unreasonable Increase of Medical Expenses in Public Hospitals(2015)* and *Suggestions on the Implementation of Comprehensive Reform of County-level Public Hospitals(2015*) issued by the government both proposed to reduce the price and income proportion of the examination and treatment of HTME in public hospitals.

The allocation level of CT and MRI scanners in Zhejiang and Guangdong in Eastern regions of China is highest among the five provinces. The different levels of socioeconomic development across China may be one of the main reasons for the results, which has been recognized by studies. The result of panel regression analysis in this research also validated the influencing effect of GDP on the distribution of CT and MRI scanners. The influence of economic factors may also lead to concentration in big cities. For instance, the percentage of CT scanners in Xi’an, which is the capital of Shannxi, reached 39.1% of the total in 2013, whereas the percentage of MRI scanners in Guangzhou, Shenzhen and Foshan reached 52.4% of the 21 cities in Guangdong. Attention should be paid to the phenomenon to avoid the over-concentration of HTME and the allocation plan of HTME formulated by the health department should ensure accessibility in poor areas.

Compared with OECD countries, the five provinces had much lower mean and median number of CT scanners per million population. The numbers of CT scanners per million population in Korea and Japan, China’s neighboring countries, are much higher than those in China. In fact, Japan has achieved over 100 CT scanners per million population. The numbers of MRI scanners per million population in the five provinces were more than 50% lower than the mean and median in OECD countries. OECD countries also gained more MRI scanners per million population than the five provinces did in 2013. These findings corroborate that the availability of CT and MRI scanners in China is far behind the average level of availability in OECD countries. However, China’s annual growth rate is much higher than the average annual growth rate in OECD countries. Given the economic development and health demands of China, the increase in CT and MRI scanners will continue and the gap will be narrowed. However, the number of CT and MRI scanners in OECD countries is just a reference for China. Quantity should not be the only priority, but the reasonable allocation level should be determined as well by combing with actual demand.

Panel regression analysis contends that the number of health professionals and the number of beds have a larger impact on distribution than other variables in the panel regression models. The influencing effect of health service indicators on the distribution of HTME has been confirmed in certain studies [12, 29, 30]. Comparison among health service indicators in our research may extend existing investigations and provide policy recommendations for the allocation plan of HTME in China. The determinants of the distribution of HTME still need further exploration and health service indicators should be involved in future research. The number of health professionals and the number of beds should be emphasized in formulating allocation plan. A formula consisting of these indicators could also be derived to determine the quantity of CT and MRI scanners.

In terms of the equity assessment of CT and MRI scanners in the five provinces, the Gini coefficient and concentration index validate that the distribution of CT and MRI scanners is fairly equitable and that the distribution of CT scanners is more equitable than that of MRI scanners. CT scanners are used more widely than MRI scanners, due to the lower price of CT scanners, making them more affordable. Thus, MRI scanners are likely to concentrate in cities with good economic condition. The equity of the distribution of CT and MRI scanners has improved after the implementation of the CON policy. The allocation plan for Type-IIHTME that is issued by the provincial health department has taken economic factors and health needs into consideration to some extent. The Gini coefficients of CT scanners validate that Shaanxi has the most equitable distribution of CT scanners, followed by Hunan, Zhejiang, Shanxi, and Guangdong. Up to 2013, the distribution of CT scanners in Shaanxi, Hunan, Zhejiang, and Shanxi had been in absolute equity, while that of Guangdong had been in relative equity. Shaanxi also had the most equitable distribution of MRI scanners, followed by Zhejiang, Hunan, Shanxi, and Guangdong. Up to 2013, the distribution of MRI scanners in Shaanxi, Hunan, and Zhejiang had been in absolute equity, while that of Shanxi and Guangdong had been in relative equity. The varying levels of socioeconomic development among cities in Guangdong may explain the low equity.

The concentration indices of CT scanners in the five provinces are all positive but no more than 0.25 and have all decreased during the period. It indicates that CT scanners used to gather in cities with good economic conditions, but the situation has improved. The concentration indices of MRI scanners are also all positive and no more than 0.30. The concentration indices in Hunan and Shanxi have decreased, whereas the rest has increased. It indicates that the distribution equity of MRI scanners based on economy is not equitable as that of CT scanners, and that MRI scanners also tend to gather in cities with good economic conditions. Meanwhile, the situation has become even worse in some provinces. The influence of economic factors accords with the regression analysis above, and the results are consistent with the findings of previous studies [16, 17].

Several policy recommendations were made on the basis of the analysis above. First, allocation plan of HTME formulated by the health department should ensure distribution equity in regions of different economic conditions. Financial subsidies and technical support should be taken to support the configuration of HTME in poor or remote areas. Second, the decision on the allocation number is based not only on population or level of other countries, but also on the actual demand of different regions. Some prediction equations should be explored to make precise decisions. Third, the allocation number of HTME could be influenced by socio-economic indicators and health service indicators. Prediction equations should involve these indicators, especially the number of health professionals and the number of beds. Lastly, the health department should prioritize the areas with large gaps of economy, such as Guangdong Comprehensive actions, including policy intervention, economic adjustment and talent support, should be adopted to fill the gaps of the allocation of HTME in these areas.

### Limitations

Given the lack of details of CT and MRI scanners in the five provinces, the distribution trends and distribution equity of the different rows of CT scanners and different tesla of MRI scanners were not analyzed in this study. The distribution equity of CT and MRI scanners at the city level was unavailable, which could therefore be accurate in the future. Moreover, only seven variables were included in the panel regression analysis, we were also unable to explore more variables relevant to the distribution of CT and MRI scanners, especially the quantity of older people. The contribution factors of distribution inequity were not explored, either. It means that we cannot provide many suggestions to improve the distribution equity. CT and MRI scanners are now common medical equipment in China. Although they are listed in the management catalogue of HTME, the real development level of HTME may not be revealed through study on them. Therefore further exploration on other HTMEs is necessary.

## Conclusions

According to the analysis above, we conclude that large gaps still exist in the distribution of CT and MRI scanners between China and OECD countries. Combined with demand, a reasonable increase in quantity is expected. In addition to GDP and population, health service indicators, especially the number of health professionals and the number of beds should also be emphasized in the allocation plan of CT and MRI scanners. Future analyses should include additional factors to inform the allocation management. The distribution equity of CT and MRI scanners in the five provinces is relatively good. However, areas with large socioeconomic disparities need considerable attention to ensure the distribution equity of CT and MRI scanners. The quantity of HTME in poor or remote areas should be guaranteed in the allocation plan. Along with the development of economy and the health service system, we have experienced a long period of mismanagement of HTME that came with rapid increase in quantity. The implementation of the CON policy helps improve the situation in China and may also help establish the scientific management system of HTME in other fast-developing countries. Moreover, the concentration of HTME in rich areas should be given significant attention and health service indicators should also be considered when formulating the allocation plan in those countries.

## Abbreviations

AGR: Annual growth rate
CMDA: Chinese Medical Doctor Association
CON: Certificate of Need
CT: Computed Tomography
DASP: Distributive Analysis Stata Package
FE model: fixed effect model
HTME: High Technology Medical Equipment
MoH: Ministry of Health
MRI: Magnetic Resonance Imaging
OECD: Organization for Economic Co-operation and Development
